# Pharmacokinetics of tulathromycin following administration to stocker cattle with remote delivery devices

**DOI:** 10.1093/jas/skz311

**Published:** 2019-10-04

**Authors:** J Daniel Rivera, Amelia R Woolums, Steeve Giguère, Joseph T Johnson, Alexis G Lutz, Paige N Tipton, William B Crosby, Ivy Hice, Merrilee Thoresen

**Affiliations:** 1 Mississippi Agriculture and Forestry Experiment Station, Mississippi State University, Poplarville, MS; 2 Department of Pathobiology and Population Medicine, Mississippi State University, Mississippi State, MS; 3 Department of Large Animal Medicine, University of Georgia, Athens, GA; 4 Deceased; 5 Department of Animal and Dairy Science, Mississippi State University, Mississippi State, MS

**Keywords:** antimicrobial, cattle, pharmacokinetics, remote drug delivery

## Abstract

Remote delivery devices (**RDD**) are used by some to administer antimicrobials (**AM**) to cattle when treatment by manual injection is logistically difficult. However, it is not clear that the pharmacokinetics (**PK**) of AM administered by RDD is comparable to that for AM administered by injection; thus, it is not certain that cattle treated by RDD experience equivalent AM effect. Fifteen crossbred beef steers (body weight [BW] = 302.5 ± 21.7 kg) were used in a three-way crossover study to determine the PK of tulathromycin following administration with RDD in the BQA injection triangle. Cattle were treated by each of three methods at 2.5 mg of tulathromycin per kg of BW with a 60 d washout period between treatments: 1) subcutaneous injection of tulathromycin (SC), 2) treatment by RDD delivered by air pump projector (AIR, Pneudart, Model 178B) at 4.5 m distance, and 3) treatment by RDD delivered by CO_2_-powered projector at 7.5 m (CO_2_, Pneudart, Model 176B). Blood was collected prior to injection and at various points up to 552 h post-administration, pharmacokinetic data were analyzed as a mixed model using animal as a random effect and method of administration, order of administration, and their interaction as fixed effects. Plasma creatine kinase (**CK**) was measured before treatment and at 24 h after treatment to determine the degree of muscle injury resulting from each treatment. Three darts administered by AIR did not discharge (20%; 95% CI = 4% to 48%); and results from these steers were excluded from analysis. Maximum plasma concentration (718, 702.6, and 755.5 µg/mL for SC, AIR, and CO_2_, respectively) and area under the concentration-time curve (17,885, 17,423, and 18,796 µg • h/mL for SC, AIR and CO, respectively) were similar and not significantly different between methods of administration. There was an effect of time (*P* = 0.0002), period (*P* = 0.0001), and interaction between method of administration and study period (*P* = 0.0210) on plasma concentration of CK. However, method of treatment (*P* = 0.6091), interaction between method and time (*P* = 0.6972), interaction between period and time (*P* = 0.6153), and 3-way interaction between method, period and time (*P* = 0.6804) were not different. Results suggest that PK of tulathromycin following delivery by RDD can be similar to subcutaneous injection; however, failure of RDD to discharge after delivery by some types of projectors can cause an important proportion of cattle to fail to receive drug as expected.

## Introduction

Remote drug delivery devices (**RDD**, aka darts) have historically been used to deliver drugs to wild mammals for immobilization (Bigalke, 1965). Despite being widely used, data suggest inconsistencies in the dosage of drug delivered by RDD (Bergerud et al., 1964). Nonetheless, RDD have been adopted for antimicrobial administration in beef cattle, particularly in stocker cattle. The use of RDD as a method of administering antimicrobials to cattle in extensive systems has become more common, in order to reduce labor demands and stress for cattle. The stocker cattle phase adds value to lightweight high-risk beef cattle who may be immunocompromised (Peel, 2003). These cattle are typically grown on pastures (Asem-Hiablie et al., 2018), and this extensive system of production may provide challenges for moving and restraining morbid cattle for antimicrobial treatment. Edwards (2010) suggested to employ low stress methods for handling morbid animals to improve their response to antimicrobial. Thus efforts to decrease stress when treating cattle for disease, as intended by some who use RDD to deliver antimicrobials, are rational. However, the National Cattleman’s Beef Association has expressed concerns regarding the use of RDD as a method to deliver antimicrobials (BQA, 2019) such as inadequate dosing of antimicrobial and muscle damage from RDD.

The research described here was undertaken to improve understanding of the effects of antimicrobial delivery by RDD on antimicrobial pharmacokinetics and muscle damage in treated cattle. The objective of this study was to determine the pharmacokinetics of tulathromycin in cattle treated by two different types of RDD, as compared to cattle treated by standard restraint and antimicrobial delivery by manual SC injection, and to assess muscle damage in treated cattle by analysis of plasma concentrations of the skeletal muscle enzyme creatine kinase (**CK**) before and after treatment. Tulathromycin was chosen because it is commonly used for treatment of bovine respiratory disease complex in stocker cattle, and it is also labeled for treatment of infectious bovine keratoconjunctivitis and infectious pododermatitis. Additionally, the relatively low injection volume of the formulation makes it feasible for administration by RDD.

## Materials and Methods

### Animals, Treatment, and Sample Collection

All protocols were approved by the Mississippi State University IACUC, Protocol #17–228. Fifteen crossbred (English × Continental) beef steers (body weight [BW] = 302.5 ± 21.7 kg) were used in a crossover design to examine the pharmacokinetics of tulathromycin delivered by three methods. All cattle had previously been halter trained to facilitate handling and sampling. The study occurred at the Mississippi Agricultural and Forestry Experiment Station – White Sand Branch Beef Unit, approximately 16 km west of Poplarville, MS. One day prior to the start of each block, cattle were weighed individually and restrained and fitted with an IV catheter placed into the jugular vein. Briefly, the site was clipped and subjected to sterile prep. The area was blocked with approximately 5 mL of 2% lidocaine, and a stab incision was made through the skin. A 14 g 13 cm IV catheter (Extended Use MILACATH #1411, MILA International, Inc., Florence, KY) was placed with an extension set with approximately 5 mL volume to facilitate blood collection. Individual BW were used to determine dosage of tulathromycin (Draxxin, Zoetis, Parsippany, NJ). In the first block, cattle were randomly assigned to one of three treatments in a crossover design (*n* = 5 animals per treatment) which would apply for the duration of the study. The tulathromycin dose was calculated at 2.5 mg/kg BW, but in order to use darts that were fully filled, as recommended by the dart manufacturer, the volume of antimicrobial administered was rounded up to the next whole mL. Treatment was given via subcutaneous injection (**SC**), delivery via air (**AIR**) pump RDD (Model 178B, Pneudart, Williamsport, PA), or delivery via CO_2_ (**CO**_**2**_) RDD (Model 176B, Pneudart). Darts used for RDD were the Type U (1.27 cm needle, 14 g) that ranged in volume from 7 to 10 mL (Pneudart). Once the correct dosage of tulathromycin was determined, each of the RDD used were weighed empty, filled with antimicrobial, weighed full, and labeled to correspond to the delivery method and the calf number. For each block, a new CO_2_ cartridge was installed in the CO_2_ RDD. Moreover, for each block cattle were administered treatments on the opposite side from where they were administered treatment in the previous block. Prior to the study, one person was designated as the administrator, and this individual practiced with the RDD devices until he felt he could comfortably administer the antimicrobial within the area of the BQA recommended injection triangle on the neck region. In addition to the RDD, this same individual applied all the SQ treatments as well. Consistent with RDD manufacturer recommendations, for the CO_2_ group the device was fired from a distance of 7.6 m, and for the AIR group the device was fired from a distance of 4.6 m after seven pumps. On day 0, cattle were restrained by tying their halters, and their respective distances were measured off using a measuring wheel (Contractor’s Measuring Wheel; Lufkin, Sparks, MD) for RDD delivery. Cattle in the SC group were simply led to a squeeze chute where they were restrained and antimicrobial was delivered using a disposable syringe and a 1.27 cm, 16 g needle.

Prior to antimicrobial delivery blood was collected in the following manner: approximately 20 mL of blood mixed with heparinized saline in the catheter and extension set was collected via syringe; following this, an additional 10 mL was withdrawn and placed into vacuum tubes containing potassium heparin for antimicrobial concentration determination. Following the 10 mL collection, the 20 mL of blood-heparinized saline mixture withdrawn first was returned via the catheter, followed by 10 mL of heparinized saline to flush the line. The 10 mL blood sample collected for antimicrobial determination was stored on ice. Following baseline blood collection, each animal was administered its respective treatment, and blood was collected in the same manner as previously described at 0.25, 0.50, 0.75, 1.0, 1.50, 2.0, 3.0, 4.0, 6.0, 8.0, 12, and 24 h. Blood samples were kept on ice no longer than 2 h. Once the dart was observed to have fallen out of the animal, it was collected, and reweighed to determine if the full dose of antimicrobial was discharged.

After IV catheters were placed and during the first 24 h blood collection period, cattle remained tied; however, they were able to lie down, they had access to water throughout the day, and soybean hulls were offered in the evening. Fans were set up to ensure the cattle stayed cool. Once the 24 h sampling was finished, the catheters were removed and the cattle were put in 1.2 ha paddocks consisting of bahiagrass close to the working facilities. Cattle had ad libitum access to water and beef mineral (Cattleman’s Edge 2:1, Free Choice Mineral, Provimi North America Inc., Brookville, OH). Further blood samples were collected at 48, 72, 96, 120, 168, 216, 264, 312, 360, 408, 456, 504, and 552 h, with an allowed margin of error of ±2 h at each sampling. Post 24 h blood collection was done via jugular venipuncture using a vacuum tube containing potassium heparin. Following the last sampling period, cattle were allowed to remain in pasture where they continued to have free-choice access to water and mineral for a 60 d washout period. After each washout period, the aforementioned processes were repeated. Block 1 began on May 30, 2017, block 2 began on July 31, 2017, and block 3 began on October 2, 2017.

Plasma was separated from blood by centrifugation at 4,900 × *g* at 4 °C for 15 min. Two 1-mL samples of plasma were placed into duplicate tubes labeled for each calf for determination of tulathromycin concentration, and 1 mL of plasma was transferred to a microcentrifuge tube for determination of CK. Samples were frozen at –80 °C until they were analyzed. This was repeated two more times for a total of three separate study blocks. Each animal was treated by each of the three methods tested; therefore, each animal served as its own control.

### Plasma Tulathromycin and CK Assay

At the completion of the study, plasma samples were shipped overnight on dry ice to the Veterinary Medicine Research and Development (VMRD) group at Zoetis for tulathromycin assay. The scientific personnel completing the tulathromycin assay were unaware of the sample treatment group designations. The analysis of tulathromycin in bovine plasma used a validated ultraperformance liquid chromatography tandem mass spectrometer (**UPLC-MS/MS**) protein precipitation extraction method. The extraction procedure was performed on a Hamilton Microstar liquid handling system (Hamilton Company, Reno, NV), or extracted manually. The sample analysis conducted was performed according to the method validated by Zoetis with the exception that the column temperature was changed from 45 °C in the validated method to 55 °C to improve the chromatography. The acquisition and peak integration data were collected in Analyst (v1.6.2, AB Sciex, Framingham, MA). Regression and concentration information was determined in Watson LIMS (v7.4.1 for Windows, Thermo Fisher Scientific, Inc., Waltham, MA).

Plasma samples were submitted to Diagnostic Laboratory at the Mississippi State University College of Veterinary Medicine for CK assay on the Axcel Clinical Chemistry System (Alfa Wassermann Diagnostic Technologies, LLC, West Caldwell, NJ).

### Pharmacokinetic Analysis

For each calf, plasma tulathromycin concentration vs. time data were analyzed based on noncompartmental pharmacokinetics using commercially available software (PK Solutions 2.0, Summit Research Services, Montrose, CO). Maximum plasma concentration (**C**_**max**_) and time to achieve maximum plasma concentration (**T**_**max**_) were obtained directly from the data. The rate constant of the terminal phase (λ _z_) was determined by linear regression of the logarithmic plasma concentration vs. time curve using a minimum of five data points. Half-life of the terminal phase (**t**_**½λz**_) was calculated as ln 2 divided by λ _z_. The area under the concentration-time curve (**AUC**) and the area under the first moment of the concentration-time curve (**AUMC**) were calculated using the trapezoidal rule, with extrapolation to infinity using C_min/λz_, where C_min_ is the plasma concentration at the last measurable time point. Mean residence time (MRT) was calculated as: AUMC/AUC. Apparent volume of distribution per fraction of dose absorbed based on the AUC (Vd_area_/F) was calculated as: IV dose /AUC• λ _z_, apparent volume of distribution per fraction of dose absorbed at steady state (Vd_ss_/F) was calculated as (IV dose/AUC)/(AUMC/AUC), and systemic clearance per fraction of dose absorbed (CL/F) was calculated from: IV dose/AUC.

### Statistical Analysis

Normality of the pharmacokinetic data and plasma CK concentrations were assessed based on examination of histograms and normal Q-Q plots of the residuals. Variance of the data was assessed by plotting residuals against predicted values. Pharmacokinetic variables were compared between methods of administration using mixed-effects linear models. Calf was modeled as a random effect to account for repeated measurements and method of administration, order of administration, and interaction between method of administration and order of administration modeled as fixed nominal effects. Model fit was assessed using Akaike’s information criterion values. The interaction term was not statistically significant (*P* > 0.05) and did not improve model fit; it was not used in the final models. *T*_max_ data was not normally distributed and analyzed using the Friedman repeated measures ANOVA on ranks. Plasma CK concentrations were transformed to the natural logarithm and analyzed using mixed-effects linear models with calf modeled as a random effect method of administration, time (baseline vs. 24 h), order of administration, and 2- and 3-way interactions modeled as fixed nominal effects. Model fit was assessed using Akaike’s information criterion values. All multiple pairwise comparisons were performed using the method of Sidak to control for family-wise type I error rates. For all analyses, *P* < 0.05 was considered statistically significant.

### Results and Discussion

All RDD were delivered within the BQA triangle recommended injection site. The AIR RDD did not deploy in 3 of 15 calves (20%; 95% CI = 4% to 48%); this was identified by weighing the RDD before they were delivered and after they dropped from the neck of cattle. The difference in weight before and after delivery of the three RDD that failed to deploy antimicrobial was less than 1 gram, while the difference in weight before and after delivery for all RDD that deployed antimicrobial ranged from 7 to 9 grams. The three calves treated with RDD that did not deploy had very low C_max_ (48, 17, and 21 ng/mL) and AUC_0–∞_ (554, 158, and 197 µg • h/mL), and plasma concentrations were below the LOQ by 24 h in two of these calves and by 72 h in the third. The CO_2_ RDD deployed in all 15 calves. Similarly, Coetzee et al. (2018) found that 4 out of 15 RDD administered with the same AIR projector used here (Pneudart Model 178B) failed in their delivery of antimicrobial. In that study, cattle treated with RDD that did not deploy were recognized by relatively little injection site reaction, compared to other treated cattle; RDD for these cattle were also weighed and found to weigh nearly the same before and after delivery. In both studies, cattle treated with RDD that did not deploy the full dose of antimicrobial were found to have low plasma concentrations of tulathromycin for 12–72 h after treatment. The failure of an important proportion of RDD to deploy in both of these studies represents a serious limitation of the use of RDD for antimicrobial delivery, at least when delivered by certain projectors. While failure of an RDD to deploy anesthetic drugs can be readily recognized when treated animals fail to become sedated, failure to deploy an antimicrobial is not evident unless the RDD is recovered and weighed, and this is unlikely to occur in most production settings. Failure of 20%–27% of cattle to receive antimicrobial as expected could obviously lead to negative effects on health and production; it also causes useless expense as the medication is left in the RDD. Additionally, the low concentrations of tulathromycin found in the plasma of cattle that did not receive a full dose indicates that a small amount of antimicrobial leaked from the needle into the cattle. While the RDD delivered by the CO_2_-powered projector (Pneudart 176B) deployed in all cases, at the time of this writing, this projector is no longer available. The difference in results obtained with the two different projectors used in this study demonstrates that results obtained with one type of RDD projector should not be extrapolated to other types of projectors. Further research is needed to determine the rates of RDD failure for different types of projectors, and the factors that influence failure rates.

**Figure 1.**
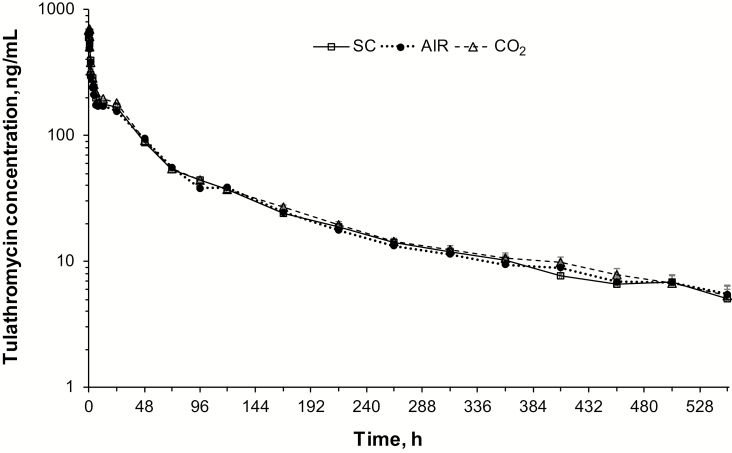
Mean plasma tulathromycin concentrations (±SD) after administration of tulathromycin by subcutaneous injection, using an air-powered dart gun, or using a CO_2_ cartridge-powered dart gun to 15 steers using a randomized Latin square crossover design. Air-powered darts did not deploy in three calves and results are excluded for these three calves.

In the study reported here, the plasma concentration vs. time data were similar for all three methods of administration when data from the three calves treated with AIR RDD that did not deploy were removed from the analysis (Figure 1). Similarly, when these three calves were excluded, pharmacokinetic variables were not different between methods of administration (*P* > 0.05 for all comparisons, Table 1). In contrast, Coetzee et al. (2018) found that the area under the plasma concentration by time curve (AUC) was lower (*P* = 0.005) for cattle receiving tulathromycin via AIR RDD compared to SC injection, and plasma clearance was increased (*P* = 0.025). The reason for the difference in our findings vs. those of Coetzee et al. (2018) are not clear, although there were some differences in study methodology that may have influenced the results. In this study, English x Continental steers were treated from a distance of 4.6 m; the cattle were tied, but standing with their necks relaxed when treated, and the hair over the injection site was not clipped before treatment. In contrast, Coetzee et al. (2018) treated Holstein steers from a distance of 9.1 m; the cattle were restrained in a chute with their heads tied to extend their necks, and the hair over the site of injection was clipped before treatment. Also, the study reported here was a crossover study, allowing each animal to serve as its own control, while in the study by Coetzee et al. (2018), the tulathromycin pharmacokinetics in 15 cattle treated by AIR RDD were compared to those in eight cattle treated by SC injection.

**Table 1.** Plasma pharmacokinetic variables (mean ± SD unless otherwise specified) after administration of tulathromycin by subcutaneous injection, using an air-powered dart gun or using a CO_2_ cartridge-powered dart gun to 15 steers using a randomized crossover design

	Method of Administration		
Variable^1^	Subcutaneous	Air-powered dart gun^2^	CO_2_ cartridge dart gun
λ _z_, h^−1^	0.0037 ± 0.0004	0.0036 ± 0.0006	0.0039 ± 0.0007
t_½λz_, h	189.1 ± 20.50	195.9 ± 33.92	185.4 ± 38.19
AUC_0–24h_, µg • h/mL	17,885 ± 3772.9	17,423 ± 4996.6	18,796 ± 4344.9
AUC_0–∞_, µg • h/mL	19,475 ± 3964.8	18,946 ± 5183.0	20,498 ± 4344.3
MRT, h	171.5 ± 17.53	176.2 ± 21.89	174.9 ± 43.60
C_max_, µg/mL	718.0 ± 405.7	702.6 ± 336.7	755.5 ± 360.4
T_max_, h^3^	0.25 (0.25 to 1.0)	0.25 (0.25 to 0.75)	0.25 (0.25 to 0.75)
Vd_area_/F, L/kg	38.3 ± 9.61	43.4 ± 18.9	35.8 ± 13.2
Vdss/F, L/kg	24.0 ± 6.04	27.1 ± 11.7	23.4 ± 9.12
CL/F, mL/h/kg	139 ± 27.8	149 ± 46.0	131 ± 27.7

^1^λ _z_ = rate constant of the terminal phase. t_½λz_ = half-life of the terminal phase. AUC_0–24h_ = Area under the plasma concentration vs. time curve from time 0 to 24 h. AUC_0–∞_ = Area under the plasma concentration vs. time curve extrapolated to infinity. MRT = Mean residence time. C_max_ = Maximum plasma concentration (observed). T_max_ = Time to maximum plasma concentration (observed) after the first dose. Vd_area_/F = Apparent volume of distribution per fraction of dose absorbed based on AUC. Vd_ss_/F = Apparent volume of distribution per fraction of dose absorbed at steady state. CL/F = Systemic clearance per fraction of dose absorbed.

^2^Excluding results from three calves in which the air-powered dart gun did not deploy.

^3^Median and range.

**Figure 2.**
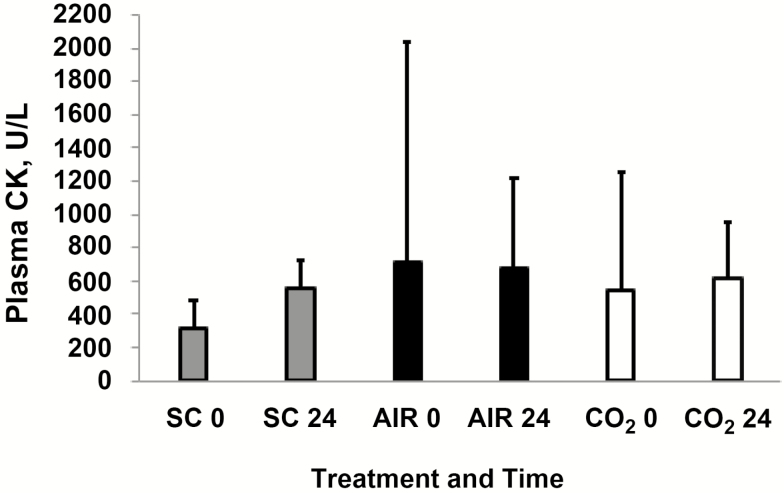
Arithmetic mean (±SD) plasma concentration of creatine kinase (CK) at time 0 and time 24 h for stocker calves treated with tulathromycin by SC injection (SC), CO_2_ projected RDD (CO_2_), or compressed air projected RDD (AIR) (normal plasma CK range = 64–405 U/L). The RDD did not discharge for three AIR calves and results from these calves are excluded, thus *n* = 12 for AIR 0 and AIR 24. For one calf, the SC and CO_2_ plasma samples were mislabeled and thus excluded, so *n* = 14 for SC 0, SC 24, CO_2_ 0, and CO_2_ 24. While there was an effect of time (*P* = 0.0002), period (*P* = 0.0001), and interaction between route of administration and study period (*P* = 0.0210) on plasma CK concentration, there was no effect of route (*P* = 0.6091), interaction between route and time (*P* = 0.6972), interaction between period and time (*P* = 0.6153), and three-way interaction between route, period and time (*P* = 0.6804).

Plasma concentrations of CK for cattle in each treatment group at time 0 and 24 h are shown in Figure 2. There was an effect of time (*P* = 0.0002), study period (*P* = 0.0001), and interaction between route of administration and study period (*P* = 0.0210) on plasma concentration of CK. However, route (*P* = 0.6091), interaction between route and time (*P* = 0.6972), interaction between period and time (*P* = 0.6153), and three-way interaction between route, period and time (*P* = 0.6804) were not different. Thus, overall, we did not identify a difference in degree of muscle injury as measured by plasma CK concentrations for cattle treated by SC injection, AIR RDD, or CO_2_ RDD, although differences might have been detected if CK had been measured at more time points, or if the area under the concentration by time curve had been assessed. Regardless of the route of administration, geometric mean CK concentrations were lower at baseline (330 U/L; 95% CI = 268 to 406 U/L) than at 24 h post-administration (536 U/L; 435 to 660 U/L), indicating that overall, treatment did cause some muscle injury, but the degree of injury was not the same in all periods, or for all routes in each period. It should be noted that for many cattle the serum CK values were not greatly elevated outside the laboratory normal range of 64–405 U/L.

Geometric mean CK concentrations in AIR calves were higher during period 1 (990 U/L; 617 to 1,587 U/L) than during period 2 (232 U/L; 145 to 372 U/L) or period 3 (335 U/L; 209 to 537 U/L). Differences in CK concentrations between treatment periods were not different for other routes of administration. Only for treatment period 1, geometric mean CK concentrations were higher for AIR calves (990 U/L; 617 to 1,587 U/L) relative to SC calves (423 U/L; 321 to 557 U/L). Differences in CK concentrations between routes of administration were not different for treatment period 2 or treatment period 3. The reason that CK concentrations were higher in AIR calves is not clear, but because values were high at baseline in AIR calves as well as at 24 h after treatment, the elevated CK could not be attributed solely to muscle injury resulting from treatment with the RDD. The elevated plasma CK in AIR calves may have been due at least in part to muscle injury when certain calves in that group were brought up from pasture and introduced into the chute for placement of the IV catheter on day 1; this seems plausible as some calves were more fractious than others. As indicated by the high SD for time 0 in the AIR group (Figure 2), the range of CK values for AIR calves at time 0 was high. The difference in plasma CK between AIR and SC calves in period 1 was driven high baseline (time 0) plasma CK values in three of five air calves in period 1 (1,126, 1,178, and 4,831 U/L). Similar to our findings, Coetzee et al. (2018) found an effect of time but no effect of treatment or treatment by time interaction on serum CK values in calves treated with AIR RDD vs. SC injection; they did, however, find significantly higher serum concentrations of aspartate aminotransferase (**AST**), another enzyme that can be released from injured muscle, at 24, 48, and 72 h post-treatment in cattle treated by AIR RDD but not at later time points.

Few reports have described work to examine the efficacy of RDD as a method to deliver antimicrobials to cattle. Van Donkersgoed et al. (1999) examined a crossbow type RDD and found no differences between RDD and hand delivery of oxytetracycline on the number of animals that had identifiable injection site reactions, the amount of tissue trim, or the number of injection sites that exceeded administrative action levels when cattle were harvested 28 d after treatment. One report recently receiving attention in the lay press evaluated the deposition of food-grade dye in tissues of cattle treated with RDD delivered with a CO_2_-powered projector and concluded that materials delivered by RDD are deposited in a manner comparable to subcutaneous (**SC**) injection (https://www.bovinevetonline.com/article/study-darts-can-reliably-deliver-sub-q-injections, accessed June 9, 2019). In contrast, Coetzee et al. (2018) compared the results of SC injection of tulathromycin to delivery by RDD with a compressed air-powered projector, and determined that cattle treated by RDD demonstrated increased signs of pain, tissue inflammation, and acute stress, compared to cattle treated by subcutaneous injection. These investigators also found that the mean area under the plasma tulathromycin concentration-time curve was lower, and the plasma clearance of tulathromycin was higher, for cattle treated by RDD, indicating decreased exposure to the antimicrobial. Perhaps most seriously, Coetzee et al. (2018), found that the RDD failed to discharge medication for 4 of 15 treated cattle.

Anecdotal reports and lay literature (e.g. https://www.bovinevetonline.com/article/study-darts-can-reliably-deliver-sub-q-injections) suggest that RDD are commonly used for administration of antimicrobials on some stocker and cow-calf operations. The research described here indicates that the pharmacokinetics of a commonly used antimicrobial can be comparable for cattle treated by RDD to those resulting from SC injection. However, it is important to note that this research did not evaluate tissue tulathromycin concentrations in cattle treated by RDD; thus, we are not able to recommend appropriate withdrawal times. Moreover, given that both the present study and that of Coetzee et al. (2018), found that at least 20% of cattle treated by RDD delivered by compressed air-powered projector failed to receive a therapeutic dose of medication, additional research should be undertaken to determine the rate of failure for different types of projectors, the factors that influence failure rates, and appropriate withdrawal times for medications commonly administered by different types of RDD and projectors.

## Conflict of Interest

In-kind support (tulathromycin assay and supplies for plasma collection) was provided by Zoetis. In order to minimize effects of conflict of interest on the research reported here, no one at Zoetis was informed of the treatment designation for plasma samples assayed for tulathromycin, and no one at Zoetis reviewed this manuscript prior to submission.

## References

[CIT0001] Asem-Hiablie S, Rotz C. A., Stout R., and Place S.. 2018 Management characteristics of beef cattle production in the eastern United States. Prof. Anim. Sci. 34:311–325.

[CIT0002] Bergerud T. A., Butt A., Russell H. L., and Whalen H.. 1964 Immobilization of Newfoundland caribou and moose with succinylcholine chloride and Cap-Chur equipment. The Journal of Wildlife Management. 28:49–53.

[CIT0003] Bigalke R. C 1965 Experiments in immobilising ungulate mammals. Zoo. Africana. 1:239–247.

[CIT0003a] BQA, 2019 National Cattleman’s Beef Association Beef Quality Assurance, National Manual. https://www.bqa.org/Media/BQA/Docs/bqa_manual_final.pdf

[CIT0004] Coetzee J. F., Kleinhenz M. D., Magstadt D. R., Cooper V. L., Wulf L. W., Van Engen N. K., Smith J. S., Rand N., KuKanich B., and Gorden P. J.. 2018 Pneumatic dart delivery of tulathromycin in calves results in lower antimicrobial concentrations and increased biomarkers of stress and injection site inflammation compared with subcutaneous injection. J. Anim. Sci. 96:3089–3101. doi:10.1093/jas/sky222. 29873747 PMC6095363

[CIT0005] Edwards T. A 2010 Control methods for bovine respiratory disease for feedlot cattle. Vet. Clin. North Am. Food Anim. Pract. 26:273–284. doi:10.1016/j.cvfa.2010.03.005. 20619184

[CIT0006] Peel D. S 2003 Beef cattle growing and backgrounding programs. Vet. Clin. North Am. Food Anim. Pract. 19:365–85, vi. 12951738 10.1016/s0749-0720(03)00032-x

[CIT0007] Van Donkersgoed J., VanderKop M., Salisbury C., Sears L., and Holowath J.. 1999 The effect of administering long-acting oxytetracycline and tilmicosin either by dart gun or by hand on injection site lesions and drug residues in beef cattle. Can. Vet. J. 40:583–587. 12001341 PMC1539762

